# Microstructure and Phase Evolution Characteristics of the In Situ Synthesis of TiC-Reinforced AZ91D Magnesium Matrix Composites

**DOI:** 10.3390/ma15041278

**Published:** 2022-02-09

**Authors:** Xinjun Zhou, Zhengfu Zhang, Xiulan Li, Liyu Zhou, Xudong Zhang, Manjiao Chen

**Affiliations:** 1 Faculty of Materials Science and Engineering, Kunming University of Science and Technology, Kunming 650093, China; zxjhhy9@163.com (X.Z.); chenmjkg@126.com (M.C.); 2 Faculty of Mechanical Engineering, Sichuan University of Science & Engineering, Yibin 644000, China; hhqlxl@163.com (X.L.); zhouliyucd@126.com (L.Z.); zhangxdqhg@126.com (X.Z.)

**Keywords:** AZ91D magnesium alloy, in situ TiC, Al_3_Ti, formation mechanism, thermodynamics, kinetics

## Abstract

TiC-reinforced AZ91D magnesium alloy composites were synthesized through the in situ reaction between an AZ91D melt and Ti-C-Al preforms. The microstructural evolution characteristics and phase transformation were investigated at different melt reaction temperatures (1013, 1033, and 1053 K), with the aim of understanding the in situ formation mechanism of TiC particles from thermodynamic and kinetic perspectives. The results showed that the temperature played a critical role in determining the formation and morphology of TiC. Initially, only the Al_3_Ti phase was formed through the reaction between Ti and Al when the temperature was 1013 K. With the increase in the melt temperature, the A1_3_Ti’s thermodynamic stability decreased, and dissolution and precipitation reactions occurred at higher temperatures (1033 and 1053 K, respectively), contributing to the formation of TiC particles. The formation of the TiC phase was attributed to two factors: Firstly, A1_3_Ti as an intermediate product reacted with carbon and formed TiC with increasing temperature. Secondly, the in situ TiC reaction was promoted due to the increased reaction-driving force provided by the increasing temperature.

## 1. Introduction

Magnesium and its alloys are used in the aviation, spaceflight, and transport industries due to their low density, high specific stiffness and strength, good damping performance, large reserves, easy recovery, and strong anti-electromagnetic interference capability [1,2,3]. Magnesium and its alloys are known as the “green engineering materials of the 21st century” [4]; however, their poor mechanical properties, low corrosion resistance, and wear resistance greatly restrict their further application. Al and Zn as basic elements are often added to pure magnesium in order to improve the corrosion and mechanical properties. However, Al and Zn have a limited effect on improving the properties of magnesium alloys; excessive amounts of Al and Zn actually lead to a significant deterioration in the material properties. Therefore, magnesium alloys with Al and Zn are always the optimal choice for improving the overall properties. Generally, the corrosion resistance of magnesium alloys is often improved via surface treatment technologies, such as laser surface texturing [5], superhydrophobic coating [6], and Cirrus HYBRID^TM^ coating technology [7], while composite materials are a more popular choice for improving the overall mechanical properties of magnesium alloys. Therefore, the development of magnesium matrix composites through the addition of a reinforcement phase to the magnesium alloy, in order to enhance its mechanical properties, has attracted the attention of material engineers and researchers.

Magnesium matrix composites have been prepared via various processing techniques, such as powder metallurgy [8,9], spark plasma sintering [10,11], semi-solid processing [12,13], solution heat treatment [14], and roll bonding [15]. Initially, the research focused on the addition of ceramic particles and oxides such as WC, TiC, SiC, TiB_2_, B_4_C, Al_2_O_3_, and MgO, with a higher strength and lower price, into magnesium alloys [16,17,18,19,20,21,22]. Later, the research on magnesium matrix composites pivoted around developing corrosion- and wear-resistant materials through the preparation of stannate, addition of graphene oxide, and ZrSiO_4_ [23,24,25,26]. TiC is considered a good potential reinforcing candidate among all of these reinforcing particles due to its unique properties, such as its low density (4.93 g/cm^3^), good thermal and chemical stability, and having the highest specific strength and specific modulus among the transition metal carbides [27]. Therefore, magnesium matrix composites reinforced with TiC particles show excellent mechanical properties, and are widely regarded as promising high-performance structural materials.

The TiC phase can exist in magnesium matrix composites through either the addition of TiC particles or an in situ synthesis reaction. Composite materials exhibit variations in their properties when subjected to different preparation processes. TiC-reinforced magnesium matrix composites prepared via powder metallurgy exhibit improved mechanical properties, as well as corrosion, friction, and wear resistance [28,29]. In contrast to the matrix, the composite materials experienced a maximum increase of 46% in their corrosion resistance, while their tensile strength, yield strength, and elongation had peak values of 861 MPa, 754 MPa, and 10.8%, respectively. Through the self-propagating high-temperature synthesis process, the prepared TiC-particle-reinforced materials exhibited an improved yield stress and an ultimate tensile strength of more than 10% and 18%, respectively, and ductility values increased by 30% [30,31]. The precipitation of nano-sized MgZn_2_ was enhanced via mechanical stirring with the ultrasonic treatment of the Mg-4Zn-0.5Ca alloy reinforced with Ti. The as-extruded composite exhibited the best performance, with a yield strength of 369.8 MPa, an ultimate tensile strength of 393.6 MPa, and an elongation of 6.7% [32]. The combination of multidirectional forging with extrusion can also initiate the precipitation of MgZn_2_ in the magnesium matrix; the TiC+MgZn_2_-reinforced magnesium matrix composites exhibited ultrahigh mechanical properties [33]. As for the additions, the wettability between the added particles and the matrix alloy, the interface stability, and the limited strengthening effect must be considered in order to further improve their properties.

The in situ synthesis technique exhibits better wettability and compatibility between the reinforcement and the magnesium matrix when compared to the direct addition of TiC particles; furthermore, the interface is cleaner. The development of TiC-reinforced magnesium matrix composites via in situ reaction also improved the damping capacity, and especially the tensile strength, at high temperatures [34]. The AZ91 magnesium alloy with in situ TiC+TiB_2_ reinforcement yielded a 47.72% increase in tensile strength, with a corresponding improvement in wear resistance [35,36,37]. The addition of the mixed SiC+TiC nanoparticles changed the Mg_17_Al_12_ morphology from plate-like to lamellar, thereby enhancing the yield strength, tensile strength, and fracture toughness [38]. In fact, these particles formed an in situ precipitation, improving the performance by refining the grain size of the magnesium alloy—especially the addition of rare-earth elements during in situ reactive infiltration [39,40]. Recent studies have focused on analyzing the mechanical properties of magnesium alloys reinforced with TiC particles. However, the TiC formation mechanism and strengthening mechanism have not been explored carefully. In this paper, TiC was synthesized in situ in the AZ91D magnesium alloy via reaction infiltration and the solid stirring method. The influence of different temperatures on the in situ formation and microstructural evolution of TiC was studied; the thermal conditions and kinetic formation process of the in situ TiC-reinforced magnesium matrix composites were investigated.

## 2. Materials and Methods

The powders selected for the experiment were Al powder (≤2 μm) (Zhongnuo New Material Technology Co., Ltd. Beijing, China), Ti powder (≤13 μm) (Zhongnuo New Material Technology Co., Ltd. Beijing, China), and C powder (≤1.5 μm) (Zhongnuo New Material Technology Co., Ltd. Beijing, China). Initially, the Ti and C powders were mixed at a molar ratio of 1:1. This was followed by adding 20 mass% Al to the mixed Ti+C powders to prepare the preform powders. Subsequently, these powders were homogeneously dispersed in acetone for 30 min via mechanical stirring together with an ultrasonic treatment. After drying in a vacuum furnace, the Ti-C-Al powders were pressed into preforms with a height of 12 mm and a diameter of 20 mm. Subsequently, the Ti-C-Al preforms were heated at 423 K for 1 h, and the furnace was cooled down to ambient temperature.

**Figure 1:**
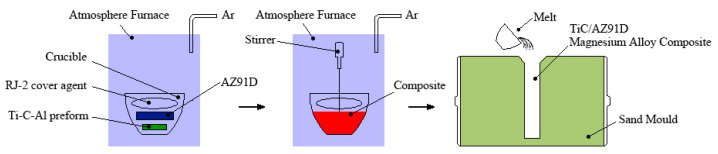
Schematic diagram showing the preparation process of the composites.

The AZ91D magnesium alloy was the substrate material, and the composites were prepared in a box-type atmosphere furnace protected by argon gas, as shown in Figure 1.

The Ti-C-Al preforms were placed at the bottom of the graphite crucible, and the AZ91D magnesium alloy was located on the preforms. Finally, an RJ-2 magnesium-alloy-covering agent was used to cover the top of the AZ91D magnesium alloy. The studied alloy was melted in graphite crucibles, and the experimental heats were recorded in a high-temperature furnace at 1013, 1033, and 1053 K. After the magnesium alloy was melted, the metal was kept liquid for 30 min. The temperature was then lowered to 833 K for the stirring treatment for 10 min. The alloy was heated to the respective melting temperature for 10 min and then cast in a sand mold. After the as-cast specimen was cooled down to room temperature, it was cleaned, cut, and polished. The hardness was measured using a Vickers diamond pyramid indenter with a load of 1.96 N with a dwell time of 15 s. The microhardness was obtained from the average of eight experimental measurements. The phases in the specimens were determined via X-ray diffraction (XRD). Meanwhile, the microstructure and composition of the phases were examined using an IE500M optical microscope (OM) and a Sigma 300 scanning electron microscope (SEM) equipped with energy-dispersive X-ray spectrometry (EDS), after etching with 4% HNO_3_.

## 3. Results and Discussion

### 3.1. Microstructure and Hardness of the TiC-Reinforced AZ91D Magnesium Matrix Composites

**Figure 2:**
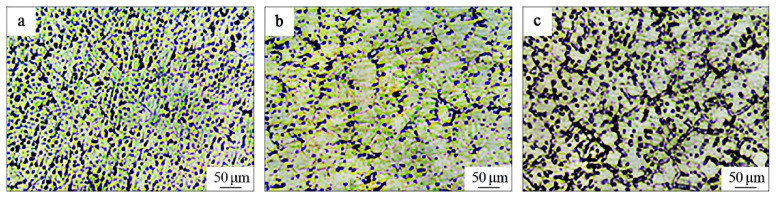
Microstructure of the magnesium matrix composites at different temperatures: (**a**) 1013, (**b**) 1033, and (**c**) 1053 K.

Figure 2 shows the microstructure of the AZ91D magnesium matrix composites with the 20% Ti-C-Al preform addition at 1013, 1033, and 1053 K.

**Figure 3:**
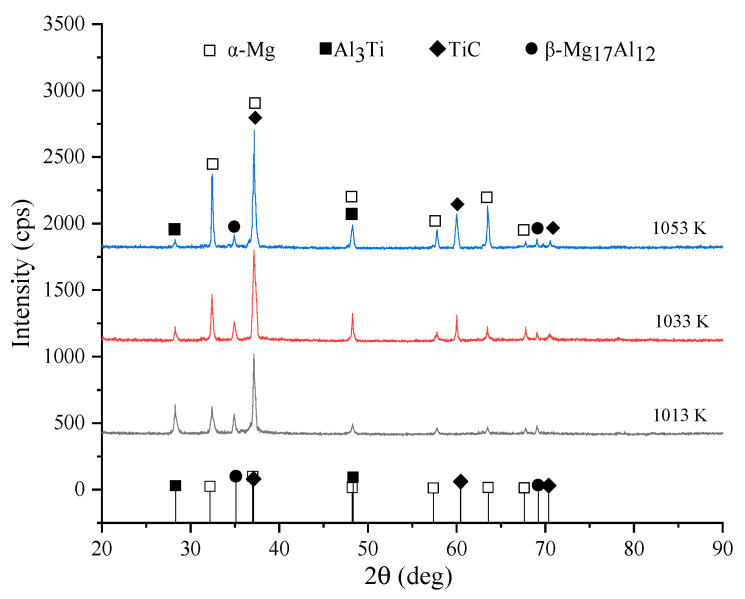
XRD patterns of the TiC/AZ91D magnesium matrix composites at different temperatures.

A white phase region was dominant in the microstructures, accompanied by reticular or semi-reticular connectors and a dark phase region at the three different temperatures. It was observed that the amount of the dark phase region in the microstructure increased as the temperature increased, with the highest amount obtained at 1053 K. Figure 3 shows the XRD analysis results of the TiC-reinforced AZ91D magnesium matrix composites treated at different temperatures. The same phases comprising α-Mg, β-Mg_17_Al_12_, and A1_3_Ti were detected in all samples, but TiC was not observed at 1013 K. The AZ91D magnesium alloy contains the α-Mg and β-Mg_17_Al_12_ phases; the β-Mg_17_Al_12_ phase usually appears at the grain boundaries of magnesium and forms a network. From the XRD pattern in Figure 3, it can be seen that the TiC peaks appeared as the treatment temperature increased to 1033 and 1053 K. Meanwhile, the peak intensity of the TiC phase increased significantly, and the A1_3_Ti phase peak decreased.

**Figure 4:**
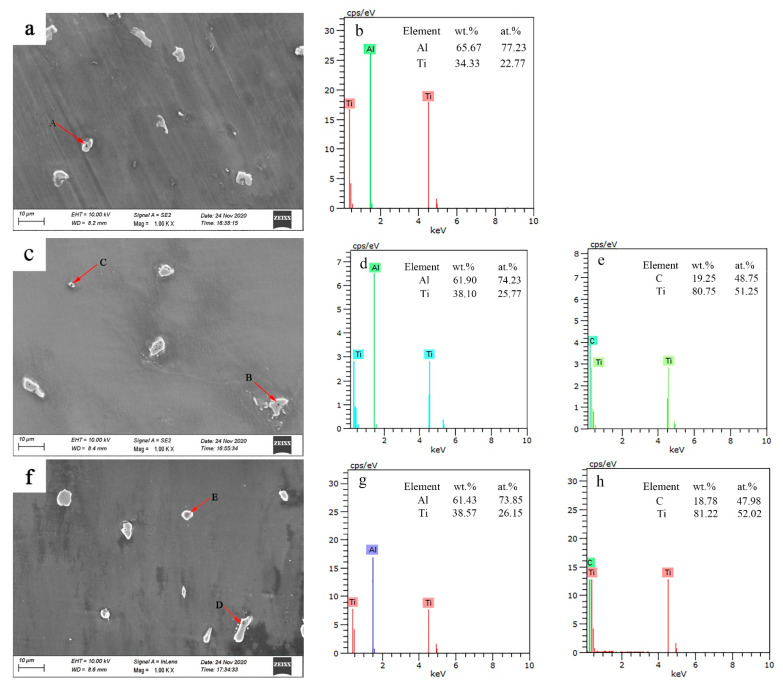
EDS analysis of the composites at different temperatures: (**a**) 1013 K; (**b**) point A; (**c**) 1033 K; (**d**) point B; (**e**) point C; (**f**) 1053 K; (**g**) point D; (**h**) point E.

In order to further confirm the phase composition and element distribution, a quantitative composition analysis of the corresponding points was performed via EDS, as shown in Figure 4.

The dark phase in Figure 3 changes as the temperature increases. According to the EDS analysis, the corresponding EDS spectrum at point A in Figure 4a revealed that these dark particles were Al_3_Ti particles with an irregular morphology. When the treatment temperature was increased to 1033 K, the EDS analysis at points B and C in Figure 4b revealed that the irregular and fine round features were Al_3_Ti and TiC, respectively. Compared with Al_3_Ti at 1013 K, the grain size of the Al_3_Ti particles decreased. As the temperature increased to 1053 K, the microstructural features became uniformly distributed in the composite, irregular features composed of the Al_3_Ti phase were observed at point D, and the TiC-rich region exhibited round and increased features at point E.

**Figure 5:**
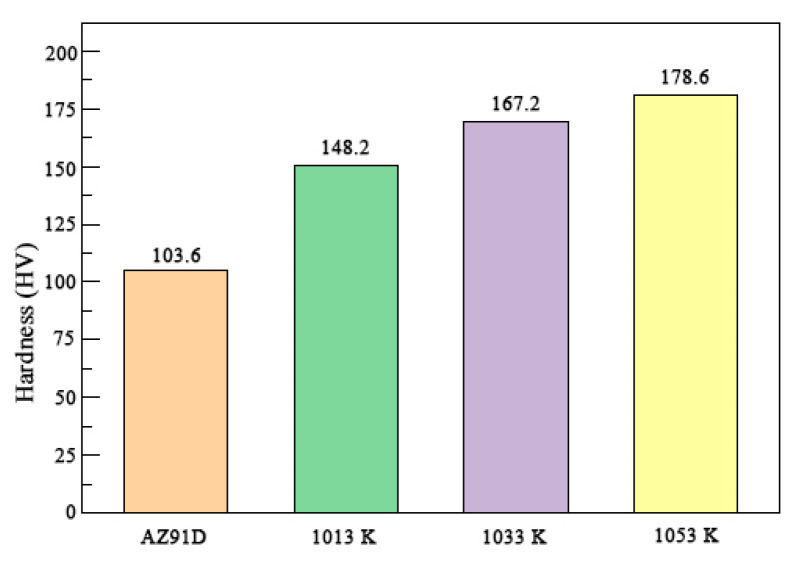
Vickers hardness of the samples with different temperatures and AZ91D contents.

Figure 5 presents the effect of temperature on the Vickers hardness of the alloys; clearly, the Vickers hardness increases with increasing temperature.

The highest hardness value was 178.6 Kgf/mm^2^, which indicates an improvement of 69% over the AZ91D matrix alloy. The variation in the hardness was attributed to the effect of TiC. The hardness values of Al_3_Ti and TiC are 700 [41] and 2800 HV [42], respectively. The increase in the amount of TiC improves the overall hardness.

### 3.2. Formation Mechanism of the In Situ TiC-Reinforced AZ91D Magnesium Alloy

#### 3.2.1. Thermal Analysis of the TiC Formation

It is necessary to ascertain whether TiC can form through the reaction between Ti and C. The change in the Gibbs free energy (∆G) of the phase reaction can determine the occurrence of a reaction. When ∆G is negative, the reaction can spontaneously occur. The smaller the negative value of ∆G, the greater the thermodynamic driving force of the reaction, which means that the reaction can easily occur. Conversely the reaction is not likely to occur when ∆G is positive. Bramfitt [43] proposed an expression to calculate the change in the Gibbs free energy, which is described as follows:

(1) $$\Delta G_{T}^{\theta }=\Delta H_{298}^{\theta }-T\Delta \Phi_{T}^{\prime }$$

where $\Delta G_{T}^{\theta }$, $\Delta H_{298}^{\theta }$, and $\Delta \Phi_{T}^{\prime }$ are the change in the Gibbs free energy, the molar entropy at 298 K, and a function of the reaction’s Gibbs free energy, respectively.

$\Delta H_{298}^{\theta }$ and $\Delta \Phi_{T}^{\prime }$ are expressed in Equation (2) and Equation (3), respectively, as follows:

(2) $$\Delta H_{298}^{\theta }=\sum {\left(n_{i}{\Delta H}_{i,298}^{\theta }\right)}_{\mathrm{products}}-\sum {\left(n_{i}{\Delta H}_{i,298}^{\theta }\right)}_{\mathrm{reactants}}$$

(3) $$\Delta \Phi_{T}^{\prime }=\sum {\left(n_{i}\Delta \Phi_{i,T}^{\prime }\right)}_{\mathrm{products}}-\sum {\left(n_{i}\Delta \Phi_{i,T}^{\prime }\right)}_{\mathrm{reactants}}$$

where i is the substance involved in the reaction, *n* is a stoichiometric coefficient, and ${\Delta H}_{i,298}^{\theta }$ is the standard thermal effect of the i substance at 298 K.

In the Mg-Al-C-Ti system, the following five reactions may occur [34]:

(4) $$3\mathrm{Al}\;(l)+\mathrm{Ti}\;(s)\to {\mathrm{Al}}_{3}\mathrm{Ti}\;(s)$$

(5) Ti (s)+C (s)→TiC (s)

(6) $${\mathrm{Al}}_{3}\mathrm{Ti}\;(s)+C\;(s)\to \mathrm{TiC}\;(s)+3\mathrm{Al}\;(l)$$

(7) $$\mathrm{Mg}\;(l)+2C\;(s)\to {\mathrm{MgC}}_{2}\;(s)$$

(8) $$2\mathrm{Mg}\;(l)+3C\;(s)\to {\mathrm{Mg}}_{2}C_{3\;}\left(s\right)$$

The thermodynamic data of the above five reactions are shown in Table 1 [44].

**Figure 6:**
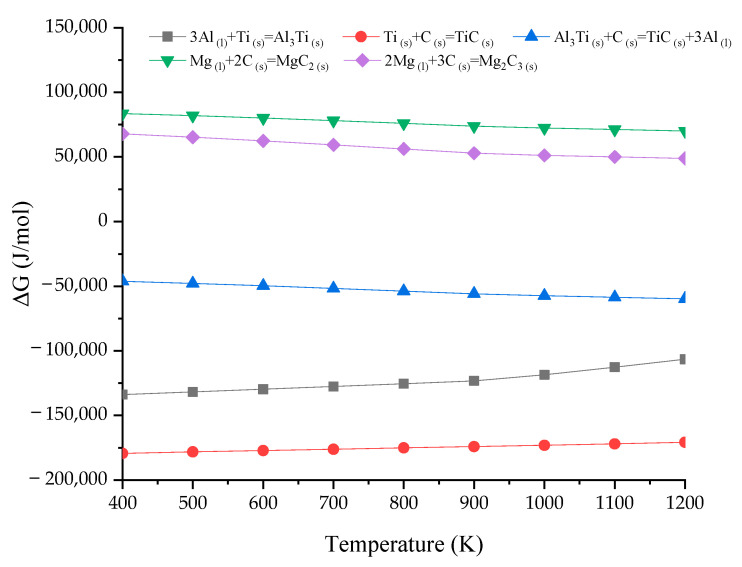
Relationship diagram between ∆G and the temperature.

Based on the above equations and the data in Table 1, the Gibbs free energy change of Equations (4)–(8) can be obtained. The relationship diagram associated with ∆G and the corresponding temperature are shown in Figure 6.

The temperatures of the experiment in this study ranged from 1000 to 1100 K; only the ∆G values of Equations (6)–(8) are negative, which means that only these three reactions can occur at these experimental temperatures. However, the ∆G value of the Al_3_Ti synthesis reaction increases with the increase in temperature; in particular, the increasing trend becomes obvious when the temperature is higher than 800 K, which indicates that Al_3_Ti as a finished product is usually thermodynamically unstable, and decomposition can occur. At the same time, the ∆G value of TiC is the lowest at each temperature, while the thermodynamic reaction-driving force increases. Meanwhile, the thermodynamic trend of the reaction between Al_3_Ti and C increases with temperature. Thus, only the formation of TiC as the final reaction product is thermodynamically favored. However, it is reasonable to state that the TiC, Al_3_Ti, and Al reaction products are generated in the microstructure. In fact, Al was not detected in the XRD pattern, indicating that the Al content is low or that Al can exist in the form of Al compounds, such as Al_3_Ti and Mg_17_Al_12_.

#### 3.2.2. Analysis of the TiC Formation Process

**Figure 7:**
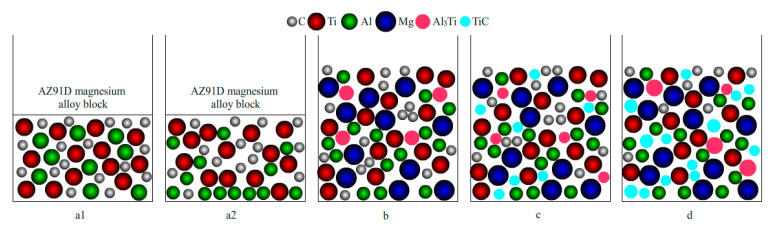
Schematic diagram of the Al_3_Ti and TiC formation processes: (**a1**) state before the Al melt and AZ91D magnesium alloy block; (**a2**) the Al powders melt and Al is located at the bottom of the crucible, but the AZ91D magnesium alloy is still in the solid state; (**b**) only Al_3_Ti is formed at 1013 K, Al+Ti→Al_3_Ti; (**c**) both Al_3_Ti and TiC exist at 1033 K; the following reactions occur: Al+Ti→Al_3_Ti, Al_3_Ti+C→Al+TiC, Ti+C→TiC; (**d**) Al_3_Ti and TiC still coexist at 1033 K, but the grain size is increased.

The benefit of thermodynamic conditions is that of indicating the possibility of a reaction; whether the reaction can take place also depends on the mobility of each element. The TiC formation process is shown schematically in Figure 7.

The Ti-C-Al preform was located under the AZ91D magnesium alloy, which caused a solid-state diffusion at the contact interface. The elemental diffusion was very slow before the melting of AZ91D, and it was considered that no reaction took place, as shown in Figure 7a1. The Al in the Ti-C-Al preform may melt when the heating temperature is higher than the melting temperature of Al. The wettability between Al and C was poor, but the wettability between Al and Ti increased with increasing temperature [45,46]. The Ti powder surface of the Ti-C-Al preform was partly enveloped by the liquid Al, which was beneficial to the formation of Al_3_Ti, but more molten Al entered the bottom of the crucible (as shown in Figure 7a2). Meanwhile, more voids occurred in the Ti-C-Al preform, and the formation of these voids was attributed to the increased fluidity of the subsequent AZ91D magnesium alloy liquid.

The AZ91D magnesium alloy melted when its melting temperature exceeded the melting point. The combined effects of gravity and the capillary force caused the molten magnesium alloy to infiltrate the voids of the Ti-C-Al preform without stirring. The molten magnesium alloy surrounded the Ti-C-Al preform. In the temperature range from 1013 to 1053 K, the superheated molten magnesium alloy increased with increasing temperature, and a different boiling occurred in the Mg-C-Al-Ti system. Thus, the molten Al at the bottom of the crucible also participated in the boiling process, which accelerated the transmission and exchange of energy between elements. The formation of Al_3_Ti between Al and Ti required an activation energy of 149 kJ/mol, while the formation of TiC between C and Ti required an activation energy of 364 kJ/mol [47]. When the energy provided by the system was less than 364 kJ/mol, Al_3_Ti was preferentially generated. Some studies have also reported [48] that Al_3_Ti can be formed when the mass fraction of Ti in Al is greater than 0.15%. In the case of the experimental temperature, Al is enriched around Ti, owing to the improved wettability between Al and Ti. Al_3_Ti was easily generated by the reaction in the microregion as long as the Al/Ti mass ratio exceeded 0.15%, as shown in Figure 7b. Arnberg [48] also pointed out that the morphology of Al_3_Ti was different at different temperatures. When the reaction temperature was greater than 1173 K, Al_3_Ti grew with a lamellar structure, also called a lamellar crystal. When the temperature was ~1023 K, the morphology of Al_3_Ti was block-shaped. At an experimental temperature of 1013 K, the energy provided by the temperature was not enough to overcome the activation energy barrier of TiC, and only Al_3_Ti existed in the microstructure, with a petal-like structure.

**Figure 8:**
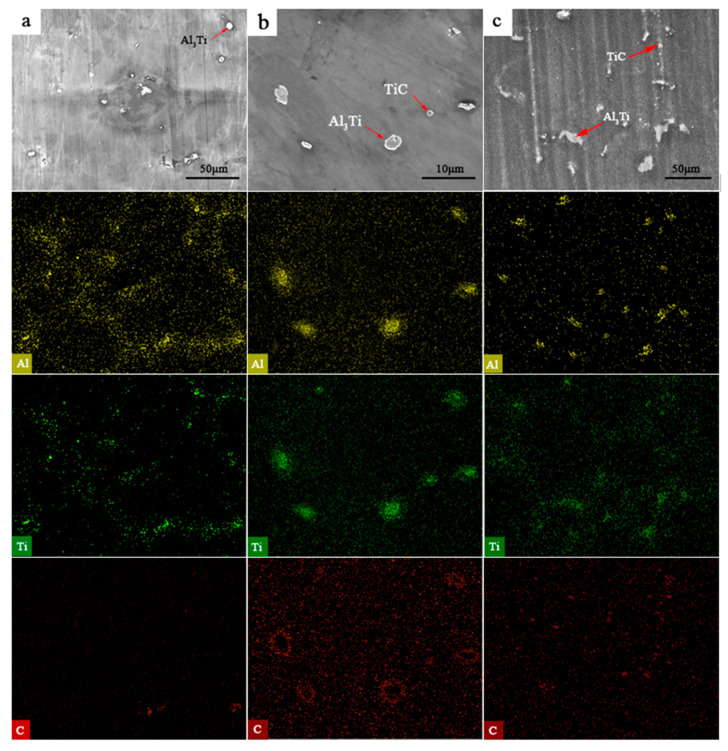
Characteristic elemental EDS-SEM mappings of the samples at (**a**) 1013 K, (**b**) 1033 K, and (**c**) 1053 K.

The EDS analysis was conducted to determine the Al, Ti, and C distributions at different temperatures. Elemental maps were acquired, and their corresponding results are illustrated in Figure 8. Ti and Al were distributed around the Al_3_Ti phase at 1013 K. It can be seen that more Ti- and Al-rich regions were located around the Al_3_Ti phase as the Al_3_Ti phase size increased, indicating that Al and Ti tended to be attracted by the growth of Al_3_Ti, resulting in the preferential growth of Al_3_Ti. However, the limited energy provided by the temperature at 1013 K was not enough to initiate Al_3_Ti coarsening. The melt was stirred continuously at 833 K for 10 min in order to attain a homogeneous distribution of the precipitates in the composites. C exhibits a partial suspension or agglomeration in melts. In addition, most C floated to the upper regions of the melts, and it was removed with dross and slag before casting.

The wettability of Ti and C improved as the temperature increased to 1033 K, enhancing the probability of the formation of the TiC particles, as shown in Figure 7c. Moreover, the in situ generation of TiC in the magnesium alloy melt depends on the external energy provided by the temperature, which should exceed the activation energy required for the reaction of Ti and C. The Ti and C contents around the in situ TiC decreased owing to the formation of TiC. The growth of TiC grains was inhibited due to the concentrations of Ti and C, thus enabling the formation of fine TiC particles. However, Al_3_Ti was readily decomposed by C due to its poor thermodynamic stability at the investigated temperatures. Thus, the following reaction occurred: Al_3_Ti+C→TiC+3Al. The enrichment adjacent to Al_3_Ti with Al and C increased the probability of the decomposition of Al_3_Ti. At the same time, more C was detected in the microstructure at 1033 K than at 1013 K (as shown in Figure 8b), with beneficial effects on the formation of TiC. However, Al_3_Ti rarely transformed into TiC due to the limited energy, so Al_3_Ti and TiC coexisted in the microstructure. The formation of TiC consumes most of the energy, and only a lower energy promotes the growth of TiC, leading to fine TiC. Meanwhile, it can be seen that the size of the Al_3_Ti phase also decreases in comparison with that obtained at 1013 K.

At 1053 K, the energy to promote the complete elimination of Al_3_Ti was limited, but the size of Al_3_Ti further decreased, and larger TiC phases were generated. In the AZ91D magnesium alloy melt at 1053 K (Figure 7d), as for Al_3_Ti, the decomposition and growth existed together. The C content around Al_3_Ti was reduced, and the decomposition of Al_3_Ti was weak, but there was more Al and Ti around Al_3_Ti (Figure 8c), which is an indication of Al_3_Ti growth. The decreased grain size of Al_3_Ti showed that the decomposition of Al_3_Ti became dominant. Ti around Al_3_Ti participated in the formation and growth of TiC; therefore, the amount of TiC adjacent to Al_3_Ti increased. In the case of TiC, more TiC was synthesized via the in situ reaction, and easily grew owing to the availability of more energy.

**Figure 9:**
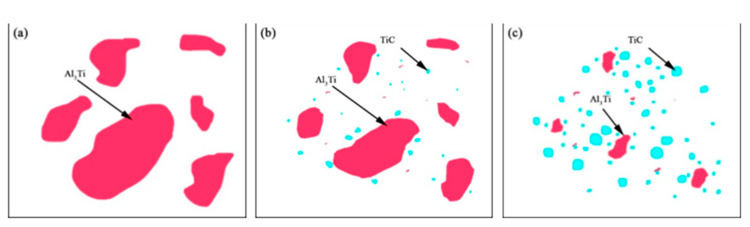
Schematic of the formation and evolution of Al_3_Ti and TiC at different temperatures: (**a**) the presence of the Al_3_Ti phase at 1013 K, (**b**) the decomposition of Al_3_Ti and formation of TiC at 1033 K, and (**c**) the final morphology and distribution of Al_3_Ti and TiC at 1053K.

In general, the microstructure, XRD patterns, and EDS analysis of the behavior of the composites can be elaborated further, as shown in Figure 9.

Figure 9 shows the schematic diagram of the microstructural evolution of Al_3_Ti and TiC at various temperatures. Initially, Al_3_Ti was distributed in the microstructure at 1013 K, as shown in Figure 9a; a small quantity of C existed in the melt because of the poor wettability between the molten matrix materials and the externally added reinforcement. The formation of TiC and the decomposition of Al_3_Ti were impeded due to the insufficient kinetics of the elemental diffusion—especially for the C elements. As the temperature increased to 1033 K, a critical C concentration at the Al_3_Ti reaction interface must be reached in order for the decomposition of Al_3_Ti to occur. The thermodynamically unfavorable Al_3_Ti overcame the activation barrier, the decomposition of Al_3_Ti occurred with an irregular morphology, and TiC was found in local areas of the Al_3_Ti decomposition. Naturally, TiC may be randomly distributed in the microstructure due to agitation. Meanwhile, TiC compounds were successfully synthesized via an in situ reactive process with the help of the thermodynamic driving force and elemental diffusion activity. The initial TiC had a smaller crystallite size. The transformation and formation processes of TiC and Al_3_Ti are described in Figure 9b. During the whole process, the wettability and diffusion of C play a major role in determining the formation of TiC and the decomposition of Al_3_Ti. The mobility of the C atoms was improved with increasing temperature. The Al_3_Ti grain size further decreased owing to the decomposition reaction by C, while the size of TiC increased, as shown in Figure 9c.

## 4. Conclusions

(1) AZ91D magnesium matrix composites reinforced with TiC were successfully synthesized using an in situ reactive infiltration technique. In the Mg-Al-Ti-C system, the Gibbs free energy change (∆G) values of the three reactions Ti+C→TiC, Al_3_Ti+C→TiC+3Al, and 3Al+Ti→Al_3_Ti were negative; thus, these reactions can spontaneously occur according to the theory. However, the ∆G of Al_3_Ti increased with increasing temperature, and became unstable. The temperature played a critical role in determining the formation of TiC. In the present experiment, the decomposition reaction of Al_3_Ti started, and the in situ reaction of TiC occurred at 1033 K;
(2) The formation mechanism of TiC in the AZ91D magnesium alloy can be described as follows: When the formation of Al_3_Ti and TiC as the reaction products is thermodynamically favored, the phase transformation driving force is promoted by the temperature. The Al_3_Ti reaction between Al and Ti first occurred when the temperature was 1013 K, due to the low energy required for the formation of Al_3_Ti. At the temperatures of 1033 and 1053 K, the energy provided by the temperature contributed to the formation of TiC via the in situ reaction between Ti and C. Meanwhile, the thermodynamically unfavorable Al_3_Ti phase decomposed into Al and TiC;
(3) In the AZ91D magnesium alloy melt, the higher the temperature, the easier the in situ TiC particle formation, with the TiC grains becoming coarser. The amount of TiC also increased as the temperature increased. The presence of TiC improved the overall hardness.

## Figures and Tables

**Table 1:** Thermodynamic data of different substances.

Substance	${\Delta H}_{i,298}^{\theta }$	$\Phi_{T}^{\prime }$								
		400 K	500 K	600 K	700 K	800 K	900 K	1000 K	1100 K	1200 K
Al_3_Ti	−142,256	98.522	106.164	114.887	123.772	132.466	140.835	148.838	156.473	163.754
C	0	6.113	6.936	7.971	9.109	10.294	11.491	12.680	13.849	14.990
TiC	−184,096	25.672	28.606	32.065	35.660	39.225	42.689	46.022	49.216	52.273
Mg_2_C_3_	75,312	104.314	112.033	120.994	130.219	139.305	148.090	156.513	164.559	172.239
MgC_2_	87,864	56.730	61.358	66.732	72.265	77.714	82.983	88.035	92.861	97.467
Mg	0	33.672	35.586	37.775	40.019	42.232	44.383	47.217	50.027	52.616
Al	0	29.301	31.192	33.355	35.570	37.756	39.888	42.724	45.679	48.382
Ti	0	31.632	33.558	35.755	38.001	40.210	42.350	44.411	46.392	48.427

## Data Availability

The raw/processed data required to reproduce these findings cannot be shared at this time, as they also form part of an ongoing study.
